# Synthesis of Highly Porous Lignin-Sulfonate Sulfur-Doped Carbon for Efficient Adsorption of Sodium Diclofenac and Synthetic Effluents

**DOI:** 10.3390/nano14161374

**Published:** 2024-08-22

**Authors:** Glaydson S. dos Reis, Sarah Conrad, Eder C. Lima, Mu. Naushad, Gopinathan Manavalan, Francesco G. Gentili, Guilherme Luiz Dotto, Alejandro Grimm

**Affiliations:** 1 Department of Forest Biomaterials and Technology, Biomass Technology Centre, Swedish University of Agricultural Sciences, SE-901 83 Umeå, Sweden; gopinathan.manavalan@slu.se (G.M.); francesco.gentili@slu.se (F.G.G.); alejandro.grimm@slu.se (A.G.); 2 Division of Geosciences and Environmental Engineering, Luleå University of Technology, SE-971 87 Luleå, Sweden; sarah.conrad@ltu.se; 3 Institute of Chemistry, Federal University of Rio Grande do Sul (UFRGS), Porto Alegre 91501-970, RS, Brazil; profederlima@gmail.com; 4 Department of Chemistry, College of Science, King Saud University, P.O. Box 2455, Riyadh 11451, Saudi Arabia; mnaushad@ksu.edu.sa; 5 Research Group on Adsorptive and Catalytic Process Engineering (ENGEPAC), Federal University of Santa Maria, Av. Roraima, 1000-7, Santa Maria 97105-900, RS, Brazil; guilherme_dotto@yahoo.com.br

**Keywords:** lignosulfonate, sustainable carbon adsorbents, heteroatom doping, lab-made effluents, pore-filling adsorption mechanism

## Abstract

Herein, a novel sulfur-doped carbon material has been synthesized via a facile and sustainable single-step pyrolysis method using lignin-sulfonate (LS), a by-product of the sulfite pulping process, as a novel carbon precursor and zinc chloride as a chemical activator. The sulfur doping process had a remarkable impact on the LS-sulfur carbon structure. Moreover, it was found that sulfur doping also had an important impact on sodium diclofenac removal from aqueous solutions due to the introduction of S-functionalities on the carbon material’s surface. The doping process effectively increased the carbon specific surface area (SSA), i.e., 1758 m^2^ g^−1^ for the sulfur-doped and 753 m^2^ g^−1^ for the non-doped carbon. The sulfur-doped carbon exhibited more sulfur states/functionalities than the non-doped, highlighting the successful chemical modification of the material. As a result, the adsorptive performance of the sulfur-doped carbon was remarkably improved. Diclofenac adsorption experiments indicated that the kinetics was better described by the Avrami fractional order model, while the equilibrium studies indicated that the Liu model gave the best fit. The kinetics was much faster for the sulfur-doped carbon, and the maximum adsorption capacity was 301.6 mg g^−1^ for non-doped and 473.8 mg g^−1^ for the sulfur-doped carbon. The overall adsorption seems to be a contribution of multiple mechanisms, such as pore filling and electrostatic interaction. When tested to treat lab-made effluents, the samples presented excellent performance.

## 1. Introduction

In recent years, the problem of water pollution caused by the increasing presence of drugs in wastewaters has raised significant concerns due to its threat to both human health and aquatic life [1,2,3,4]. Drugs such as antibiotics, analgesics, and painkillers enhance the quality of life for humans and animals, but their incomplete metabolism results in the excretion of part of these substances into the environment, where they accumulate in soil and water bodies [2,3,4]. Therefore, communal and industrial wastewaters must be properly treated before being discharged into the environment.

Conventional wastewater treatment plants are not designed to efficiently remove contaminants like drugs; thus, these end up contaminating rivers, lakes, and groundwater. Numerous methods exist for wastewater treatment, with the most common being biological processes [5,6], nanofiltration [6,7], photodegradation [8], and advanced oxidation processes [9]. While these methods are widely used globally, most have several drawbacks, including low efficiency in removing pharmaceuticals, generation of by-products and sludge, high costs, and complex operation.

Adsorption, a well-established and effective method, is used to remove pollutants that conventional wastewater treatment methods cannot easily eliminate [10,11,12]. This technique offers several advantages, including an easy implementation and operation system, low cost due to the use of inexpensive adsorbents, and high sustainability, as adsorbents can be regenerated and reused multiple times.

Activated carbon (AC) is the most employed adsorbent due to its suitable physicochemical characteristics, such as a high specific surface area and a large amount of functional surface groups, which result in excellent adsorptive properties. Activated carbon can be easily prepared from all types of biomass. Moreover, AC can be further modified through heteroatom doping (such as O, N, S, B, and P) to further improve its physicochemical properties and adsorptive performances [11,13,14].

Among heteroatom dopants, sulfur is particularly advantageous due to its natural abundance, low toxicity, low cost, and ability to enhance the microstructure and surface properties of the materials boosting its adsorptive performance. The incorporation of heteroatoms such as sulfur into the carbon network imparts unique properties that make it particularly effective in capturing and immobilizing ions and molecules in water treatment processes. Sulfur possesses an electronegativity of 2.58, close to that of carbon (2.55), which does not cause a high imbalance in the electronegativity of the sulfur-doped material, but it changes the electron distribution, leading to the creation of defects in the carbon lattice [13,15]. Such defects act as active adsorption sites, which boost the adsorptive ability of the adsorbent [15,16]. Moreover, sulfur doping may create new sulfur states in the carbon network, such as thiophene, which increases the surface reactivity and also boosts its adsorptive properties.

As far as we know, there are very few works dedicated to understanding the effect of sulfur doping on the adsorption of pollutants from aqueous solutions. Wang et al. [17] prepared sulfur-doped biochar using a banana pseudo-stem as a carbon precursor and sodium thiosulfate (Na_2_S_2_O_3_) as a sulfur dopant. The materials were employed as adsorbents for the removal of tetracycline from aqueous solutions. The authors found that the sulfur-doped carbon removed 93.89% of tetracycline, 3.11 times higher than the removal efficiency of the undoped carbon material. This increase was explained due to a larger presence of defect vacancies (that serve as active adsorption sites) on the sulfur-doped material’s structure/surface, which were generated by the introduction of sulfur atoms into the carbon matrix. In another work, Vigneshwaran et al. [18] employed tapioca peels to produce a doped carbon adsorbent using Na_2_S_2_O_3_ as a sulfur dopant. The material was tested as an adsorbent to remove two organic dyes, malachite green (MG) and rhodamine B (RhB). The results showed good adsorption efficiency of the sulfur-doped material, reaching adsorption capacities of 53.35 and 40.86 mg g^−1^ for MG and RhB, respectively. The authors reported that the sulfur-doped carbon had much better adsorptive performance because sulfur incorporated new functionalities and adsorptive sites that boosted the adsorption of the molecules by electrostatic attractions and hydrogen bonding interactions, which are responsible for boosting the overall adsorption performance.

In this research, we provide a straightforward strategy to produce highly porous sulfur-doped carbon with an extremely high surface area using lignin-sulfonate as a carbon precursor. This approach involves a one-step pyrolysis at a temperature of 700 °C after the addition of pure sulfur as the dopant and ZnCl_2_ as a chemical activator. This strategy resulted in highly porous carbon with an SSA of 1758 m^2^ g^−1^ compared to 753 m^2^ g^−1^ (non-doped carbon) and with a high amount of mesopores, highlighting the obvious improvement of the porosity imparted by the sulfur doping. To compare the effectiveness of the sulfur doping on the adsorptive features of the materials, the sulfur-doped and non-doped carbons were employed to adsorb sodium diclofenac (DFC) from aqueous solutions. The results demonstrated that the sulfur-doped carbon presented a far better efficiency in removing DCF and also in treating lab-made effluents containing several drugs. This study proves that highly porous carbon structures can be prepared using a sulfur doping strategy, with huge potential for applications not only in the adsorption of contaminants from wastewaters but also in sustainable energy technologies.

## 2. Materials and Methods

### 2.1. LS-Carbon Synthesis

The lignin sulfonate (LS) utilized in this investigation was sourced from a sulfite pulping industry (Domsjö Fabriker, Domsjö, Sweden). Elemental sulfur and zinc chloride of analytical grade were purchased from Sigma Aldrich. The lignin sulfonate carbon materials were synthesized via a one-step pyrolysis activation process [16]. Initially, LS (15 g) was mixed with ZnCl_2_ using a weight ratio of 1:3 (biomass: ZnCl_2_), and approximately 30.0 mL of water was added during blending to ensure uniform paste formation (sample named LS-carbon). For the sulfur-doped sample (LS-sulfur carbon), the same procedure was employed but with the addition of 7.5 g of pure sulfur to the paste until homogenously mixed. Subsequently, the paste of the two samples underwent drying in an oven set at 105 °C for 24 h followed by pyrolysis in a reactor externally heated by an electric furnace. The pyrolysis process was conducted at a constant heating rate of 10 °C/min, up to 700 °C under inert atmosphere for 1 h for both LS-carbon and LS-sulfur carbon samples. Afterward, the reactor was turned off and allowed to cool down to room temperature. To eliminate residual ZnCl_2_ and ashes in the two samples, the pyrolyzed samples underwent a reflux process for 2 h at 75 °C with 100 mL of a 1.0 M HCl solution. Subsequently, it was washed with distilled water until the wash water achieved the same pH as the distilled water (6.0).

### 2.2. Characterization of Activated Biochars

The textural characterization of the LS-carbons was performed by N_2_ adsorption−desorption analysis using a Tristar 3000 apparatus, Micrometrics Instrument Corp., Norcross, GA, USA. The samples were first degassed at 110 °C for 3 h under N_2_ flow and then measured at liquid N_2_ temperature (−196 °C). The specific surface area (SSA) of the samples was calculated by multipoint N_2_ sorptiometry using the Brunauer–Emmett–Teller (BET) principle. In addition, pore volume, average pore size, and pore size distribution were obtained from sorption isotherms using the Barrett–Joyner–Halenda (BJH) model.

A scanning electron microscope (SEM) Zeiss Merlin FEG-SEM instrument (Oberkochen, BW, Germany) with an in-lens secondary electron detector was used for the analysis of the morphological characteristics. The instrument was operated at an accelerating voltage of 5 kV and probe current of 100 pA. Samples were attached to carbon tape mounted on aluminum stubs and coated with 2 nm of platinum using a Quorum Technologies Q150T ES device, Lewes, UK.

Raman spectra were collected using a Bruker Bravo handheld spectrometer (Bruker, Ettlingen, Germany) attached to a docking measuring station. The LS samples were manually ground using an agate mortar and pestle, placed in 5 mL glass vials, and scanned in the 300–3200 cm^−1^ spectral range at 4 cm^−1^ resolution for 256 scans. Min–max normalization over the 1000–2000-cm^−1^ region and smoothing (9 points) was done using the built-in functions of the OPUS software (version 7, Bruker Optik GmbH, Ettlingen, Germany); baseline correction was not needed.

The surface elemental composition and surface functionalities were assessed using X-ray photoelectron spectroscopy (XPS). XPS spectra were collected using a Kratos Axis Ultra DLD electron spectrometer (Kratos Analytical, Manchester, UK), using a monochromated Al Kα source operated at 150 W. An analyzer pass energy of 160 eV for acquiring survey spectra and a pass energy of 20 eV for individual photoelectron lines were used. The samples were gently hand pressed using a clean Ni spatula into a special powder sample holder. Because carbon materials are conductive, no charge neutralization system was used. The binding energy (BE) scale was calibrated following the ASTM E2108 and ISO 15472 standards [19,20]. Processing of the spectra was accomplished with the Kratos software.

The point of zero charge (pH_pzc_) of the LS carbons was determined by adding 20.00 mL of 0.050 mol L^−1^ NaCl with a previously adjusted initial pH (the initial pH (pH_i_) values of the solutions were adjusted from 2.0 to 10.0 by adding 0.10 mol L^−1^ of HCl and NaOH) to several 50.0 mL Falcon tubes containing 30.0 mg of each LS carbon material [14]. The suspensions were shaken for 4 h at room temperature. Afterward, the suspensions were centrifuged at 5000 rpm for 10 min to separate the LS carbon from the aqueous solution. The pH_i_ values of the solutions were accurately measured using the solutions that had no contact with the LS carbons; the final pH (pH_f_) values of the supernatant after contact with the solid were recorded [14]. The value of pH_pzc_ is the point where the curve of ΔpH (pH_f_ − pH_i_) versus pH_i_ crosses a line equal to zero [14].

### 2.3. Adsorption Experiments

Adsorption of DCF from the aqueous solution was carried out in batch mode at 298 K using aliquots of 20.00 mL of DCF with initial concentrations varying from 50.00 to 1200.0 mg L^−1^ and 30.0 mg LS-based adsorbents at a pH ranging from 2.0 to 10.0. Adsorption kinetics and equilibrium were employed using nonlinear fitting, and the model’s details are shown in the Supplementary Materials. The calibration curve (Figure S1) and the analytical control concerned with the determination of DCF is given in detail in the Supplementary Materials. The statistical analysis of the adsorption models was performed based on values of R^2^_adj_ and standard deviation (SD) values (see Supplementary Materials). The information about the lab-made effluents is also shown in the Supplementary Materials.

## 3. Results and Discussion

### 3.1. Physicochemical Characterization of the LS-Based Carbons

**Figure 1:**
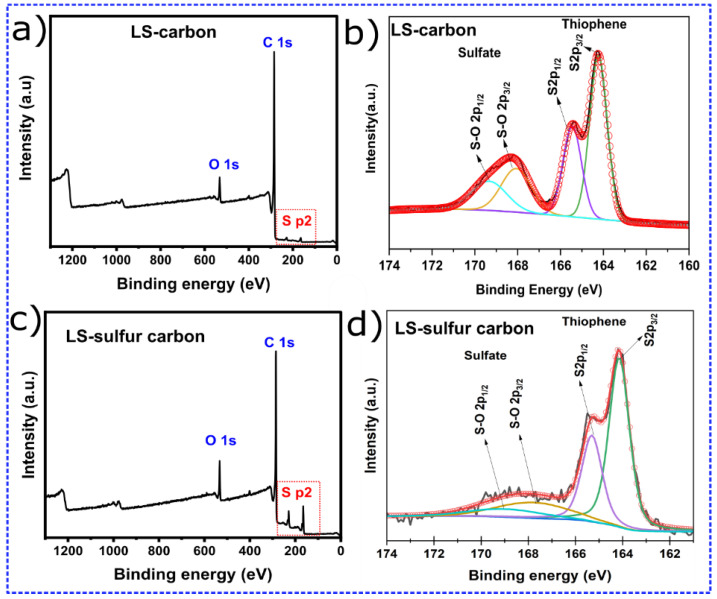
(**a**,**c**) XPS survey spectra for LS-carbon and LS-sulfur carbon samples and (**b**,**d**) deconvoluted S p2 peaks.

The LS-based carbon samples were subjected to XPS analysis to examine the influence of S doping on the surface chemistry of the materials [12,13,18]. The XPS survey spectra of LS-carbon (Figure 1a) and LS-sulfur carbon (Figure 1c) revealed that the sulfur doping successfully incorporated sulfur atoms in the LS-sulfur carbon structure due to the presence of higher peaks regarding S p2 compared to LS-carbon sulfur peaks, which were smaller due to the inherent S presence in the lignin-sulfonated precursor. Besides sulfur, carbon C1 (~284.0 eV) and oxygen O1 (~530.4 eV) peaks were also observed in both samples.

The sulfur chemical states in LS-carbon (Figure 1b) and LS–Sulfur–Carbon (Figure 1d) were analyzed by high-resolution XPS of the S 2p region. The deconvoluted spectrum displays two distinct doublets corresponding to different sulfur states. The intense peaks located at approximately 164.0 eV (S 2p3/2) and 165.2 eV (S 2p1/2) are assigned to the thiophenic sulfur species, which arise from C–S bonding within aromatic carbon networks. The observed spin–orbit splitting (Δ ≈ 1.16 eV) and the area ratio (≈2:1) are characteristic of a single sulfur state, confirming the presence of sulfur in the form of thiophene-like moieties in the carbon matrix. These thiophenic S sites are known to improve the electronic conductivity and enhance redox activity due to their ability to delocalize π-electrons within the carbon lattice. In addition to thiophenic sulfur, a second pair of deconvoluted peaks centered around 168–170 eV is observed, which is attributed to sulfate (–SO_4_^2−^). The appearance of these sulfate-related peaks indicates partial surface oxidation of the materials’ surfaces. Such oxidized sulfur species can enhance the hydrophilicity of the carbon surface, promoting better contact between solid–liquid interface and better molecules accessibility through diffusion during adsorption processes.

**Figure 2:**
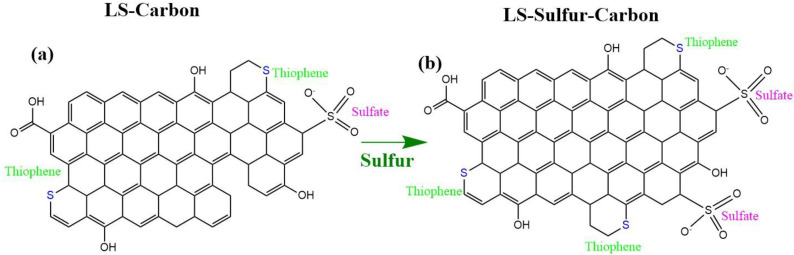
Proposed carbon network structures: (**a**) LS-carbon and (**b**) LS-sulfur carbon samples.

Together, these results confirm the coexistence of both covalently bonded (thiophenic) and oxidized (sulfate) sulfur species in both carbon structures. However, the content of sulfur functionalities present in the LS-sulfur carbon was found to be larger compared to the LS-carbon (schematic representation in Figure 2), where sulfur added during the activation process interacts with the lignin to form stable C–S linkages while also generating surface sulfate functionalities. As expected, the LS-Sulfur carbon sample presented more sulfur content due to the doping process (see Figure 2). From the XPS analysis, the atomic percentages of the elements C, O, and S were calculated using their respective peak areas after deconvolution.The atomic percentages in LS-carbon were 79.5, 3.6, and 4.1% for carbon, oxygen, and sulfur, respectively, while in LS-sulfur carbon, samples were 74.7, 4.5, and 10.1% for carbon, oxygen, and sulfur, respectively. These results highlight that the sulfur doping more than doubled the presence of sulfur atoms in the LS-sulfur carbon compared to LS-carbon. The sulfur doping also slightly increased the oxygen fixation in the carbon structure, and the higher presence of sulfur and oxygen in LS-sulfur carbon means a greater abundance of functional groups that can boost the adsorptive properties of the material.

**Figure 3:**
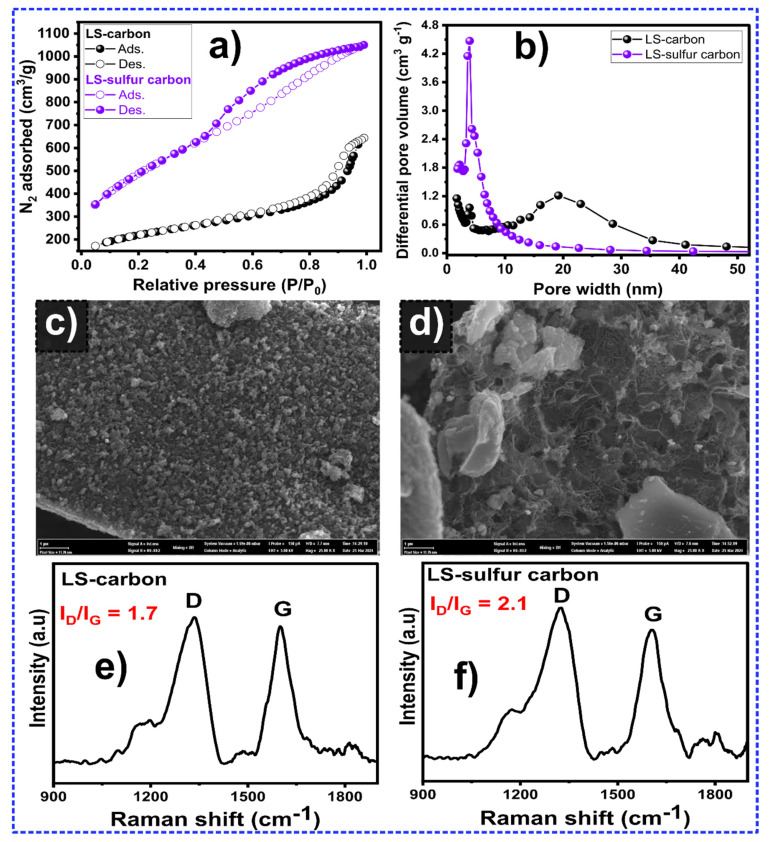
(**a**) N_2_ isotherm curves for LS-carbon and LS-sulfur carbon, (**b**) pore size distribution curves, (**c**) SEM image of LS-carbon, (**d**) SEM image of LS-sulfur carbon, (**e**) Raman spectrum of LS-carbon, and (**f**) Raman spectrum of LS-sulfur carbon.

The porosity characteristics of the adsorbent materials, such as SSA and pore size, play an important role in the materials’ ability to adsorb a wide range of pollutants in contaminated waters [21]. Figure 3a shows the N_2_ adsorption isotherms of the LS-carbon materials. Different N_2_ adsorption curves were observed as a consequence of the sulfur doping process. It seems that the sulfur-doped material exhibited an isotherm more like that of type IV [22,23], with a highlighted hysteresis, suggesting mesoporous features. Compared to the LS-carbon, the LS-sulfur carbon showed a more prominent hysteresis, indicating a larger number of mesoporous features. However, the LS-sulfur carbon curve also indicated a high presence of micropores and small mesopores due to the higher adsorbed amounts of N_2_ at low partial pressures [22,23].

The pore size distribution of LS-based carbons derived from the N_2_ adsorption isotherms are shown in Figure 3b. As shown in the figure, the LS-sulfur sample exhibited a higher presence of smaller pores (in the range of 2.0 nm to 10 nm) compared to the LS-carbon sample, which exhibited big portions of pores in the size of 15 to 30 nm. As a result of the higher number of small pores, the LS-sulfur carbon exhibited a much higher specific surface area value (1760 m^2^ g^−1^) compared to LS-carbon (753 m^2^ g^−1^), showing that the sulfur doping successfully enhanced the SSA value, which could lead to a positive impact on the LS-sulfur carbon adsorptive properties since the adsorption ability of adsorbents is positively influenced by the SSA and pore size.

The LS-carbon (Figure 3c) and LS-sulfur carbon (Figure 3d) materials’ textural properties were further examined by SEM, and important differences in their morphologies could be observed. It seems that the sulfur doping provoked a remarkable impact on the morphology of the LS-sulfur carbon sample (Figure 3d), exhibiting a more irregular structure with many different sized cavities and holes with higher apparent porosity. LS-carbon displayed what seemed to be a more intact high rugosity but no apparent cavities and holes on its surface (Figure 3c). Similar reports have addressed that the sulfur doping of biomass-based carbon can increase the number of physical defects, as observed in the SEM image, and structures richer in defects can positively impact the ability of LS-sulfur carbon to adsorb molecules since defects can act as adsorptive sites [10,13].

The impact of sulfur doping on the microstructure of the LS-carbon was further evaluated by Raman spectroscopy analysis (Figure 3e,f). Taking into consideration that the degree of structural defects may have an important influence on the adsorptive performances of the carbon material, the Raman spectrum of a biomass-carbon material provides two important main peaks, at around 1500–1650 cm^−1^, which is related to the G band (graphitic structure), and at around 1300 cm^−1^, which is related to the degree of structural defects in the carbon lattice (D band) [22,23,24]. An I_D_/I_G_ ratio can be calculated from these peaks, which indicates the material’s degree of graphitization [22,23,24]. Figure 3e,f shows that both I_D_/I_G_ values were higher than 1, suggesting the greater presence of defective carbon lattice structures in both LS-carbons; however, the sample doped with sulfur exhibited a higher I_D_/I_G_ value, which suggests that the sulfur doping increased the number of defects, which can be one of the reasons for the improved adsorptive performance due to the fact that defects can act as active adsorption sites that help to boost the material’s adsorptive properties [12,13].

### 3.2. Adsorption Experiments

#### 3.2.1. pH_pzc_ and the Effect of the pH on DCF Removal

**Figure 4:**
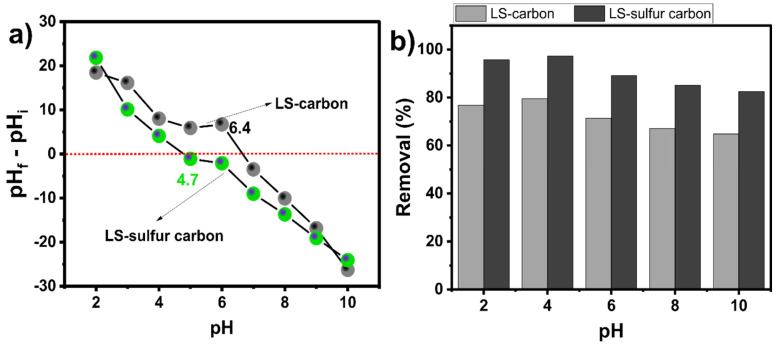
(**a**) pH_pzc_ of the LS-carbon and LS-sulfur carbon samples and (**b**) effect of the pH on DCF removal.

The pH_pzc_ of an adsorbent surface is an important parameter that plays a crucial role in the adsorption process. The pH_pzc_ indicates the point at which the adsorbent material’s surface is null: the values under the pH_pzc_ mean that the material has a positively charged surface and above pH_pzc_ is negatively charged. The pH_pzc_ of LS-carbon was 6.4 and LS-sulfur carbon was 4.7 (Figure 4a), which indicates a more acidic character of LS-sulfur carbon compared to LS-carbon, indicating that sulfur doping incorporated positive functionalities (sulfur functional groups) on LS-sulfur carbon’s surface. At a pH higher than these values, the material’s surface would have negative charges [25].

The role of pH on DCF removal may play a significant role due to the presence of different functional groups on the LS-carbon materials’ surfaces as well as the ionization of the DCF molecule. Figure 4b shows that the pH slightly affected the DCF removal on both samples. For both adsorbents, the removal efficiency of DCF slightly decreased from pH 2 to 10 (see Figure 4b). The decrease in the DCF adsorption took place under an alkaline pH, perhaps due to the fact that under this pH, both LS-carbon materials and DCF were negatively charged, provoking electrostatic repulsion between equally charged surfaces of the LS materials and DCF [12,13]. The variation in the DCF adsorption removal with the pH suggests that the electrostatic interaction between DCF and LS-carbon adsorbents played a role in the adsorption process since the DCF removal was dependent on the pH of the solution [26,27,28].

#### 3.2.2. Kinetic Studies

**Figure 5:**
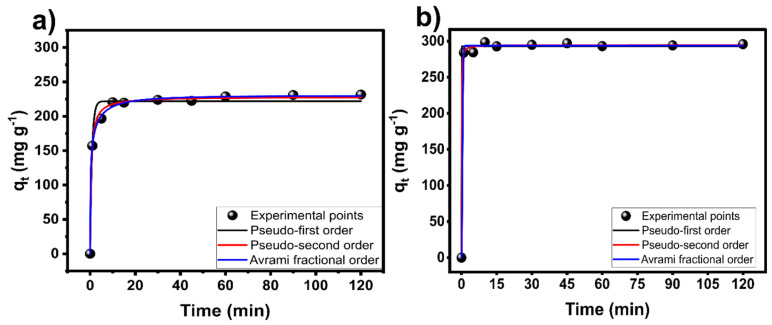
Kinetic measurements for the adsorption of DCF onto (**a**) LS-carbon and (**b**) LS-sulfur carbon samples. Initial pH of DCF solution was 6.0.

The kinetics of adsorption is a crucial study for better understanding the adsorption process because it gives important insights into how quickly pollutants are removed, thus helping to better design real adsorption systems. Figure 5 shows the DCF kinetic measurements at 298 K for the LS-carbon and LS-sulfur carbon at the initial concentration of 500 mg g^−1^. Firstly, it can be seen that the kinetics for LS-sulfur carbon seemed to be much faster than LS-carbon, which could be associated with the higher SSA and amount of surface functionalities of the sulfur-doped carbon, which also led to a higher adsorption capacity of the LS-sulfur carbon compared to LS-carbon.

Further, the kinetic process was evaluated using the nonlinear pseudo-first-order (PFO), pseudo-second-order (PSO), and Avrami fractional order (AFO) kinetic models, see Figure 5. The kinetic parameters are depicted in Table 1. The suitability of the models was judged based on R^2^_adj_ and SD [11,12,13]. Thus, AFO was the most suitable kinetic model because it presented the lowest SD and highest R^2^_adj_ values. The Avrami kinetic model equation features the Avrami exponent (n_Av_), a fractional number that signifies possible variations in the adsorption mechanism during the adsorption process [28,29]. As a result, the adsorption mechanism can adhere to multiple kinetic orders that change as the adsorbate interacts with the adsorbent [28,29].

#### 3.2.3. Isotherm Studies

The equilibrium (isotherms of adsorption) of an adsorption system is vital to evaluate the relationship between the adsorbate (DCF) and adsorbents (LS-carbon materials). Besides, it gives valuable insights into the efficiency of an adsorbent because it gives its maximum adsorption capacity [12,13,14,30,31].

**Figure 6:**
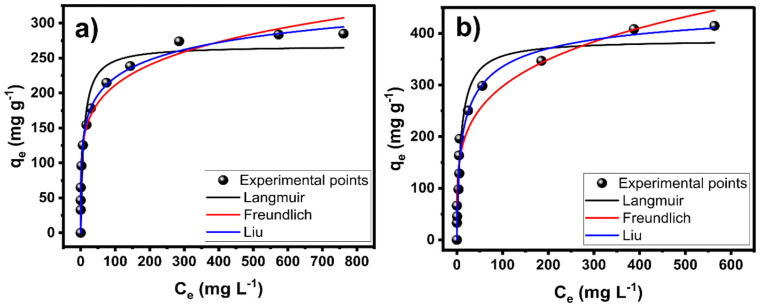
Isotherm curves for adsorption of DCF onto (**a**) LS-carbon and (**b**) LS-sulfur carbon samples. Initial pH of DCF solution was 6.0.

The adsorption isotherms of LS-carbon and LS-sulfur carbon adsorbents are shown in Figure 6. The experimental points were fitted using the nonlinear Langmuir, Freundlich, and Liu models. The fitting accuracy of the models followed the same logic of kinetic studies (based on the R^2^_adj_ and SD). Based on the results from Table 2, Liu presented the best fit because it gave the lowest SD and highest R^2^_adj_ values among the three models. The Liu model assumes that the active sites responsible for the adsorption of the adsorbate molecules/anions do not have the same energy, which means that the adsorbents (LS-carbon materials) may have active adsorption sites that the DCF molecules prefer to occupy [12,31,32]. However, the saturation of the adsorbents (total occupation of the active sites) occurred in this work at the studied conditions. Moreover, it is worth mentioning that LS-sulfur presented a far better performance for adsorbing DCF (maximum adsorption capacity of 473.8 mg g^−1^) compared to LS-carbon (301.6 mg g^−1^). Such a difference in their performances could be related to the much higher SSA value of LS-sulfur carbon as well as the larger presence of functional sulfur surface groups provoked by the sulfur doping.

The adsorption data, undoubtedly, has proven the outstanding performance of LS-sulfur carbon in removing DCF from aqueous solutions. To better assess the LS adsorbents’ performance, they were compared with other carbon-based adsorbents reported in the literature. Therefore, the q_max_ of DCF adsorption for different adsorbents are listed in Table 3. Thus, LS-sulfur carbon exhibited the highest adsorptive performance among all adsorbents listed Table 3. Such outstanding performances can be attributed to the very high surface area of sulfur-doped carbon as well as its abundance in surface functional groups, as discussed earlier in this paper. The results strongly suggest that sulfur doping is an excellent strategy for removing drugs from polluted wastewaters.

#### 3.2.4. Lab-Made Effluent Treatment Tests

**Figure 7:**
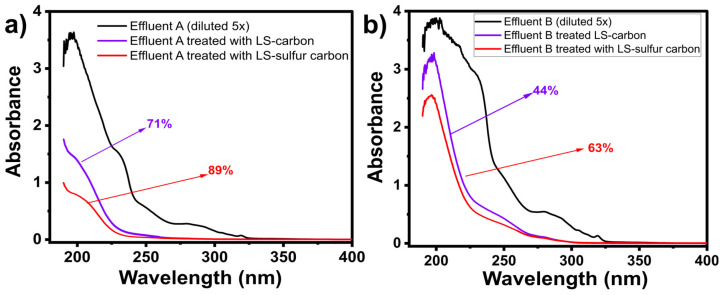
Adsorption of lab-made drug effluents. (**a**) Effluent A; (**b**) Effluent B.

The LS-carbon materials showed outstanding efficiency in removing DCF from aqueous solutions. Thus, it can be expected that these adsorbents could also be highly efficient in treating lab-made effluents containing various drugs and other organic and inorganic compounds (simulating real effluents). Two lab-made effluents were prepared (see their compositions in Supplementary Table S1). The applicability of the LS-carbon materials in treating these affluents were tested, and their results are shown in Figure 7.

The calculation of how many compounds were removed from the effluents by the LS-carbon materials is given in percentage of removal, taking into account the UV–vis spectra area of the two lab-made effluents before and after the treatment [11,21,38,39]. The results (Figure 7) showed that LS-sulfur removed far more compounds (89% and 63%) than LS-carbon (44% and 71%) from the lab-made effluent A (low concentration) and lab-made effluent B (high concentration), respectively. The higher LS-sulfur carbon efficiency in treating both lab-made effluents is in accordance with the previous adsorption tests, highlighting the excellent strategy of sulfur doping to boost the adsorptive performance of the adsorbent for removing drugs from wastewater. It is worthwhile to point out that since both carbon materials were able to reduce the pollutant levels in highly concentrated lab-made effluents as well as the DCF concentration levels, it is safe to state that both LS samples could be used to treat effluents and polluted waters containing drugs to reduce their concentrations to safe levels.

## 4. Conclusions

Waste biomass is a suitable raw material for material synthesis, with different potential applications. This work evaluated the potential application of lignin-sulfonate for synthesizing sulfur-doped carbon materials for the removal of diclofenac from water and a mixture of organic contaminants from lab-made effluents. The physicochemical characterization data strongly indicated that sulfur doping remarkably impacted the doped carbon structure. Firstly, the SSA of the sulfur-doped carbon (1758 m^2^ g^−1^) was much higher compared to that of the non-doped carbon (753 m^2^ g^−1^). Secondly, the sulfur-doped carbon exhibited more sulfur states/functionalities than non-doped, highlighting the successful incorporation of sulfur atoms in the carbon network. Thirdly, such physicochemical modification yielded a sulfur-doped material with far better adsorptive performance on removing diclofenac and contaminants from lab-made effluents. The adsorption experiments indicated that the kinetics were better described by the Avrami fractional order model with much faster kinetics for the sulfur-doped carbon. The equilibrium studies indicated that the Liu model gave the best fitness, with the maximum adsorption capacity being 301.6 mg g^−1^ for non-doped and 473.8 mg g^−1^ for the sulfur-doped carbon. The overall adsorption seemed to be a contribution of multiple mechanisms, such as pore filling and electrostatic interaction. When tested to treat lab-made effluents, the samples presented excellent performance.

## Figures and Tables

**Table 1:** Kinetic model parameters for DCF adsorption.

	LS-Carbon	LS-Sulfur Carbon
Pseudo-first order		
q_1_ (mg g^−1^)	221.9	292.7
k_1_ (min^−1^)	1.215	55.32
R^2^_adj_	0.9791	0.9969
SD (mg g^−1^)	10.32	5.146
Pseudo-second order		
q_2_ (mg g^−1^)	228.24	294.3
k_2_ (g mg^−1^ min^−1^)	0.008960	0.08191
R^2^_adj_	0.9950	0.9878
SD (mg g^−1^)	5.060	3.534
Avrami		
q_AV_ (mg g^−1^)	229.8	293.8
k_AV_ (min^−1^)	1.438	1.19
n_AV_	0.3681	6.916
R^2^_adj_	0.9972	0.9979
SD (mg g^−1^)	3.753	2.212

**Table 2:** Isotherm equilibrium model parameters for DCF adsorption.

	LS-Carbon	LS-Sulfur Carbon
Langmuir		
q_e_ (mg g^−1^)	267.8	387.8
k_1_ (L mg^−1^)	0.1125	0.1124
R^2^_adj_	0.8977	0.9417
SD (mg g^−1^)	32.21	34.70
Freundlich		
k_F_ ((mg g^−1^) (mg L^−1^)^−1/nF^)	90.31	102.1
n_F_ (dimensionless)	5.414	4.307
R^2^_adj_	0.9814	0.9329
SD (mg g^−1^)	13.74	37.23
Liu		
Q_max_ (mg g^−1^)	301.6	473.8
Kg (L mg^−1^)	0.00703	0.04805
n_L_ (dimensionless)	0.3091	0.5647
R^2^_adj_	0.9921	0.9602
SD (mg g^−1^)	8.910	28.67

**Table 3:** Comparison of the q_max_ for DCF on different adsorbents.

Adsorbents	q_max_ (mg g^−1^)	Adsorbent Dosage (g L^−1^)	T (°C)	pH	Ref.
Tree waste carbon	355	1.0	25 °C	6.0	[10]
Tree waste selenium-doped carbon	434	1.0	25 °C	6.0	[10]
H_3_PO_4_ activated hydrochar	377.99	1.5	15 °C	2.0	[33]
MnFe_2_O_4_/biochar composite	344.26	0.15	25 °C	4.0	[34]
Aquatic plant-based carbon	23.25	1.5	20 °C	6.0	[35]
Al(III)-based MOF (MOF-303)	334.89	1.0	25 °C	7.0	[36]
Al(III)-based MOF (DUT-5)	103.36	1.0	25 °C	7.0	[36]
Commercial gelatin/CNT beads	26.97	2.0	25 °C	8.1	[37]
RCTLW gelatin/CNT beads	20.57	2.0	25 °C	8.1	[37]
MgAl-biocarbon composite	168.04	4.0	60 °C	5.0	[38]
LS-carbon	301.6	1.5	25 °C	5.0	This work
LS-sulfur carbon	473.8	1.5	25 °C	5.0	This work

## Data Availability

The data will be shared upon request.

## Supplementary Materials

The following supporting information can be downloaded at: https://www.mdpi.com/article/10.3390/nano14161374/s1, Section S1. Calculation of the adsorption capacity (q) and percentage of removal (%), Figure S1. DCF calibration curve, Section S2. Models of kinetics, isotherms, and statistical evaluation, Table S1. Compositions and concentrations of effluents containing drugs and other common water pollutants.
