# Correction: dos Reis et al. Synthesis of Highly Porous Lignin-Sulfonate Sulfur-Doped Carbon for Efficient Adsorption of Sodium Diclofenac and Synthetic Effluents. *Nanomaterials* 2024, *14*, 1374

**DOI:** 10.3390/nano15241860

**Published:** 2025-12-11

**Authors:** Glaydson S. dos Reis, Sarah Conrad, Eder C. Lima, Mu. Naushad, Gopinathan Manavalan, Francesco G. Gentili, Guilherme Luiz Dotto, Alejandro Grimm

**Affiliations:** 1 Department of Forest Biomaterials and Technology, Biomass Technology Centre, Swedish University of Agricultural Sciences, SE-901 83 Umeå, Sweden; gopinathan.manavalan@slu.se (G.M.); francesco.gentili@slu.se (F.G.G.); alejandro.grimm@slu.se (A.G.); 2 Division of Geosciences and Environmental Engineering, Luleå University of Technology, SE-971 87 Luleå, Sweden; sarah.conrad@ltu.se; 3 Institute of Chemistry, Federal University of Rio Grande do Sul (UFRGS), Porto Alegre 91501-970, RS, Brazil; profederlima@gmail.com; 4 Department of Chemistry, College of Science, King Saud University, P.O. Box 2455, Riyadh 11451, Saudi Arabia; mnaushad@ksu.edu.sa; 5 Research Group on Adsorptive and Catalytic Process Engineering (ENGEPAC), Federal University of Santa Maria, Av. Roraima, 1000-7, Santa Maria 97105-900, RS, Brazil; guilherme_dotto@yahoo.com.br

## Error in Figure

In the original publication [1], there was a mistake in Figure 1d as published. Figure 1d was incorrect and has been replaced with the correct one. A mistake was made by the first author while documenting/fitting the XPS results on OriginLab software (Origin 2024b (10.15)), and the wrong spectrum was processed. The error has been corrected, and the updated Figure 1d appears below. To guarantee the accurateness of the analysis, the XPS for Figure 1c,d was re-done. In addition, Figure 1b was also updated. Figure 1b previously presented the fitting peaks between 162 and 167 eV; now it shows the complete fitting from 160 to 174 eV. Subsequently, Figure 2 was also updated, and appears below.

**Figure 1:**
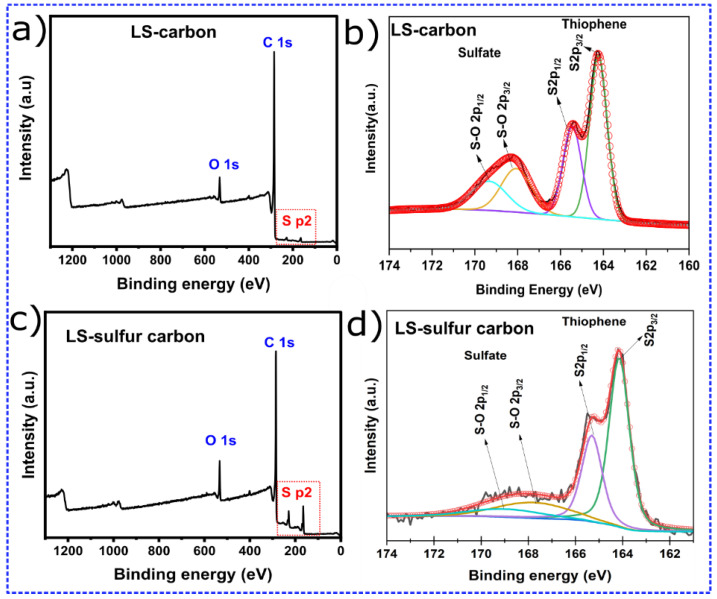
(**a**,**c**) XPS survey spectra for LS-carbon and LS-sulfur carbon samples and (**b**,**d**) deconvoluted S p2 peaks.

**Figure 2:**
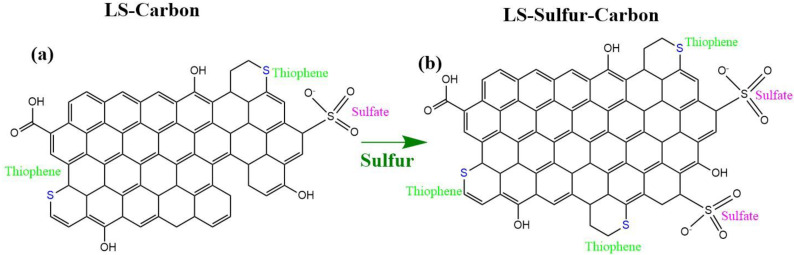
Proposed carbon network structures: (**a**) LS-carbon and (**b**) LS-sulfur carbon samples.

## Text Correction

A correction has been made to 3.1. Physicochemical Characterization of the LS-Based Carbons, Paragraphs 2 and 3: New text:

The sulfur chemical states in LS-carbon (Figure 1b) and LS–Sulfur–Carbon (Figure 1d) were analyzed by high-resolution XPS of the S 2p region. The deconvoluted spectrum displays two distinct doublets corresponding to different sulfur states. The intense peaks located at approximately 164.0 eV (S 2p_3_/_2_) and 165.2 eV (S 2p_1_/_2_) are assigned to the thiophenic sulfur species, which arise from C–S bonding within aromatic carbon networks. The observed spin–orbit splitting (Δ ≈ 1.16 eV) and the area ratio (≈2:1) are characteristic of a single sulfur state, confirming the presence of sulfur in the form of thiophene-like moieties in the carbon matrix. These thiophenic S sites are known to improve the electronic conductivity and enhance redox activity due to their ability to delocalize π-electrons within the carbon lattice. In addition to thiophenic sulfur, a second pair of deconvoluted peaks centered around 168–170 eV is observed, which is attributed to sulfate (–SO_4_^2−^). The appearance of these sulfate-related peaks indicates partial surface oxidation of the materials’ surfaces. Such oxidized sulfur species can enhance the hydrophilicity of the carbon surface, promoting better contact between solid–liquid interface and better molecules accessibility through diffusion during adsorption processes.

Together, these results confirm the coexistence of both covalently bonded (thiophenic) and oxidized (sulfate) sulfur species in both carbon structures. However, the content of sulfur functionalities present in the LS-sulfur carbon was found to be larger compared to the LS-carbon (schematic representation in Figure 2), where sulfur added during the activation process interacts with the lignin to form stable C–S linkages while also generating surface sulfate functionalities. As expected, the LS-Sulfur carbon sample presented more sulfur content due to the doping process (see Figure 2). From the XPS analysis, the atomic percentages of the elements C, O, and S were calculated using their respective peak areas after deconvolution.The atomic percentages in LS-carbon were 79.5, 3.6, and 4.1% for carbon, oxygen, and sulfur, respectively, while in LS-sulfur carbon, samples were 74.7, 4.5, and 10.1% for carbon, oxygen, and sulfur, respectively. These results highlight that the sulfur doping more than doubled the presence of sulfur atoms in the LS-sulfur carbon compared to LS-carbon. The sulfur doping also slightly increased the oxygen fixation in the carbon structure, and the higher presence of sulfur and oxygen in LS-sulfur carbon means a greater abundance of functional groups that can boost the adsorptive properties of the material.

We also state that reference [21] was wrongly cited in the original publication and therefore it was removed.

The authors state that the scientific conclusions are unaffected. This correction was approved by the Academic Editor. The original publication has also been updated.
