# Supplementary material for: Synthesis of Highly Porous Lignin-Sulfonate Sulfur-Doped Carbon for Efficient Adsorption of Sodium Diclofenac and Synthetic Effluents
Source: Nanomaterials (Basel). 2024 Aug 22;14(16):1374. doi: 10.3390/nano14161374 (PMC11357084; doi:10.3390/nano14161374)
Supplement: Supplementary file 1 [file nanomaterials-14-01374-s001.zip › nanomaterials-3118144-supplementary.pdf]

Supplementary Material for

Synthesis of Highly Porous Lignin-Sulfonate Sulfur-Doped Carbon for Efficient Adsorption of Sodium Diclofenac and Synthetic Effluents

* Corresponding author: glaydson.simoes.dos.reis@slu.se

S1. Calculation of the adsorption capacity (q) and percentage of removal (%)

The adsorption capacity as a function of time (q_t, mg g^{-1}), adsorption capacity in the equilibrium (q_e, mg g^{-1}), and removal of DCF (%) were determined by Equations S1 to S3, respectively:

$$q_t = \frac{(C_0 - C_t)}{m} V \qquad (S1)$$

$$q_e = \frac{(C_0 - C_e)}{m} V \qquad (S2)$$

$$\text{Removal (\%)} = 100 \left(\frac{C_0 - C_e}{C_0}\right) \qquad (S3)$$

Where C_0 is the initial DCF concentration in the liquid phase (mg L^{-1}), C_t is the DCF concentration at time t (mg L^{-1}), C_e is the DCF concentration at equilibrium (mg L^{-1}), m is the amount of adsorbent (g), and V is the volume of solution (L).

All the experiments were carried out in triplicate to ensure reproducibility, reliability, and accuracy of the experimental data. The relative standard deviations of all measurements were below 5%. Blanks were run in parallel and corrected when necessary.

The solutions of DCF were stored in glass bottles that were cleaned by immersing them in 1.4 mol L^{-1} HNO3 for 24 h, rinsing them with deionized water, dried, and stored in a suitable

cabinet. Standard DCF solutions (5.0-85.0 mg L^{-1}) were used for calibration in parallel with a blank. The linear analytical calibration curve was performed on the UV-Win software of the T90+ PG Instruments spectrophotometer. All the analytical measurements were carried out in triplicate, and the precisions of the standards were better than 3.1% (n=3). The detection limit of acetaminophen was 0.15 mg L^{-1} with a signal/noise ratio of 3. A 30.0 mg L^{-1} of standard DCF solution was used as quality control after every ten measurements to ensure the accuracy of the DCF measurements.

S2. Models of kinetics, isotherms, and statistical evaluation

Kinetic models

The kinetic models of pseudo-first-order (PFO, Eq. S1), pseudo-second-order (PSO, Eq. S2), and Avrami fractional order (AFO, Eq. S3) were used to adjust the experimental data, according to Equations S4 to S6, respectively.

$$q_t = q_1 (1 - \exp(-k_1 t)) \tag{S4}$$

$$q_t = \frac{k_2 . q_2^2 . t}{1 + q_2 . k_2 . t} \tag{S5}$$

$$q_t = q_{Av} (1 - \exp(-k_{Av} t)^{n_{Av}}) \tag{S6}$$

Where k_1 (min^{-1}), k_2 (g mg^{-1} min^{-1}), and k_{Av} (min^{-1}) are the pseudo-first-order, pseudo-second-order, and Avrami kinetic rate constants, respectively, q_1, q_2, and q_{Av} are the theoretical values for adsorption capacity (mg g^{-1}), t is time and n_{Av} the exponent in Avrami's model.

Isotherm models

The adsorption equilibrium data were fitted according to the non-linear Freundlich (Eq. S7), Langmuir (Eq. S8), and Liu (Eq. S9) isotherm models.

$$q_e = K_F C_e^{1/nF} \quad (S7)$$

$$q_e = \frac{q_{max} K_L C_e}{1+(K_L C_e)} \quad (S8)$$

$$q_e = \frac{Q_{max}(K_g C_e)^{nL}}{1+(K_g C_e)^{nL}} \quad (S9)$$

Where k_F is the Freundlich constant (mg g^{-1})(mg L^{-1})$^{-1/nF}$, $1/n_F$ is the heterogeneity factor, q_m is the maximum adsorption capacity (mg g^{-1}), k_L is the Langmuir constant (L mg^{-1}), k_g is the Liu equilibrium constant, q_{max} is the maximum adsorption capacity of the Langmuir or Liu model (mg g^{-1}), and n_L is the Liu exponent.

Statistical evaluation of the fitted models

The parameters of the kinetic and adsorption equilibrium models were determined by non-linear regression through the minimization of the least squares function using the Quasi-Newton method. Calculations will be performed using the Origin software (OriginPro 2016 SR0). The adequacy of the kinetic and equilibrium models was statistically assessed employing the adjusted determination coefficient (R^2_{adj}) and the standard deviation of residues (SD) shown in equations S10 and S11 below.

$$R^2_{adj} = 1 - (1 - R^2).\left(\frac{n-1}{n-p-1}\right) \quad (S10)$$

$$SD = \sqrt{\left(\frac{1}{n-p}\right).\sum_{i}^{n}\left(q_{i,exp} - q_{i,model}\right)^2} \quad (S11)$$

Where $q_{i,\ model}$ is the individual model sorption capacity expected by the model; $q_{i,\ exp}$ is the individual experimentally measured sorption capacity; $\overline{q}_{i,exp}$ is the average of all measured experimental sorption capacities; n is the number of experiments performed; p is the number of model parameters.

The R^2_{adj} and SD values were used to compare kinetics and equilibrium models. The best-fitted model would present the R^2_{adj} closer to 1.00 and the lowest SD values.

Table S1. Compositions and concentrations of effluents containing drugs and other common water pollutants

	**Concentration (mg L^{-1})**	
**Pharmaceuticals**	Effluent A	Effluent B
Acetylsalicylic acid	10	20
Propranolol	10	20
Amoxicillin	10	20
Captopril	10	20
Nimesulide	10	20
Diclofenac	10	20
Acetaminophen	20	40
**Sugars**		
Saccharose	30	50
Glucose	30	50
**Organic**		
Urea	10	20
Citric acid	10	20
Humic acid	10	20
**Inorganics**		
Ammonium phosphate	20	30
Ammonium chloride	20	30
Sodium sulfate	10	20
Sodium chloride	50	70
pH	6	6

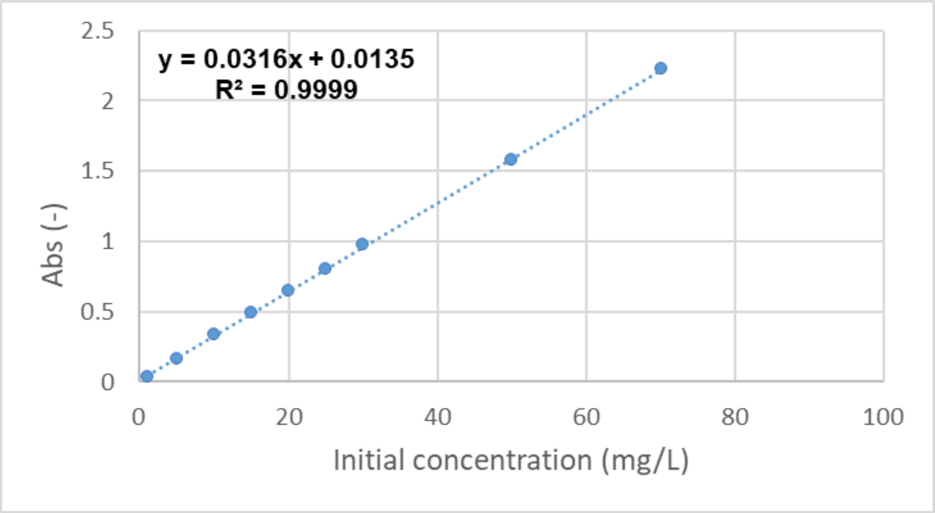

Figure S1. DCF calibration curve
